# Induced Abortion Practices in an Urban Indian Slum: Exploring Reasons, Pathways and Experiences

**Published:** 2015-09

**Authors:** Deepanjali Behera, Shalini Bharat, Nilesh Chandrakant Gawde

**Affiliations:** School of Health System Studies, Tata Institute of Social Sciences, Mumbai, India

**Keywords:** Induced abortion, Urban slum, Contraception, Religious beliefs, India

## Abstract

**Objective:** To explore the context, experiences and pathways of seeking abortion care among married women in a minority dominated urban slum community in Mumbai city of India.

**Materials and methods:** A mixed-method study was conducted using a systematic random sampling method to select 282 respondents from the slum community. One fifth of these womenreported undergoing at least one induced abortion over past five years. A quantitative survey was conducted among these women (n = 57) using structured face-to-face interviews. Additionally, in-depths interviews involving 11 respondents, 2 community health workers and 2 key informants from the community were conducted for further exploration of qualitative data.

**Results:** The rate of induced abortion was 115.6 per 1000 pregnancies in the study area with an abortion ratio of 162.79 per 1000 live births. Frequent pregnancies with low birth spacing and abortions were reported among the women due to restricted contraception use based on religious beliefs. Limited supportfrom husband and family compelled the women to seek abortion services, mostly secretly, from private, unskilled providers and unregistered health facilities. Friends and neighbors were main sources of advice and link to abortion services. Lack of safe abortion facilities within accessible distance furtherintensifies the risk of unsafe abortions.

**Conclusion:** Low contraception usage based on rigid cultural beliefs and scarcely accessible abortion services were the root causes of extensive unsafe abortions.Contraception awareness and counseling with involvement of influential community leaders as well as safe abortion services need to be strengthened to protect these deprived women from risks of unwanted pregnancies and unsafe abortions.

## Introduction

Despite a relatively liberal abortion law in India after the establishment of Medical Termination of Pregnancy (MTP) Act in 1971, high incidences of unsafe abortions still take place across the nation. This silent pandemic further adds to the existing burden of maternal morbidity and mortality in a developing country situation. About 56 percent of the total 6.4 million induced abortions are reported to be unsafe in India (1). Post-abortion complications cost around 8 percent maternal deaths annually (2). These adverse consequences are preventable through contraception practice and provision of safe abortion services (3, 4), however, the country still struggles to achieve these goals. India has the highest number of women with unmet need for contraception (5) with only 56.3 percent women practicing any contraception method (6). In contrast to, a higher acceptance for female sterilization, most birth spacing methods are not widely opted by Indian women (7). This contributes to a high level of unwanted pregnancies, many of which end in abortions (4, 8). Further, the country has absolutely inadequate number of registered abortion facilities and skilled abortion providers (9) which compels many Indian women to seek unsafe abortion methods and services.

Urban slum population is documented to have higher rates of unmet needs for family planning (10), and hence higher vulnerability to induced abortion practices. In addition, there is absolute scarcity of available reproductive services including abortion reported in urban Indian slums (11, 12). Few studies have been conducted in Indian slums to assess the abortion related practices. Mumbai, the capital city of Maharashtra state has the largest slums in India. A recent study in a suburban slum community in Mumbai illustrated an induced abortion ratio of 85.95 per 1000 live births (13). Further, previous studies undertaken in various parts of India suggest varied contexts, pathways and preferences for seeking abortion by women which give rise to diverse experiences and consequences related to abortion (1, 3, 5, 7, 14-17).

This paper examines induced abortion practices in a low income, minority dominated slum community in Mumbai city; a less explored theme in existing literature. A mixed method approach was used to assess the abortion incidence and to analyze the context and pathways of seeking abortion among women in a slum community dominated by Muslims, a majority of whom were also migrants to the city. This paper also attempts to understand the experiences of induced abortions among slum dwelling Indian women in a context of limited access to formal health services in an urban poor setting.

## Materials and methods

The study was conducted in a low income community located in a suburban slum of Mumbai city. With approximately 1400 households the study had a total population of about 8500 people (18). Majority of the population belonged to Muslim religion and were native residents of northern states of Uttar Pradesh and Bihar. Being close to a dumping ground, the site had unhygienic living conditions making the local community highly susceptible to a number of health related ailments. In addition to low literacy and lack of awareness about preventive health behaviors, the community was also deprived of adequate access to public health services. A recent community based survey report of the site revealed low contraceptive usage with various religious mis-beliefs (19). About 37 percent of married women in this area had no knowledge about any of the contraception methods. Among the 63 percent who were aware only 21 percent reported practicing any form of contraception (19). 

The study used a mixed method study design employing both quantitative and qualitative research methods. Data collection was undertaken during February-March 2012 among the married women aged 15-44 years living in the community for more than five years. After complete house listing, systematic random sampling was used to select a total of 282 households. Only one eligible woman per household was randomly selected as respondent. When selected household did not have an eligible woman or declined to participate, the next household was approached.

Quantitative data were collected from all 282 women using a pre-structured interview tool about their demographic and socioeconomic characteristics, obstetric history of past five years and the current contraception behavior. Those women with a history of induced abortion in the past 5 years were asked further questions related to abortion seeking and experience. One-fifth of the women (n = 11) with the abortion history were randomly selected for collection of qualitative data through in-depth interviews. Four key informant interviews were also conducted with two community level health workers and two volunteers from the community.

Pretested structured interview schedule was used as the instrument for quantitative data collection while interview guidelines were used during in-depth as well as key informant interviews for collection of qualitative data. All interviews were carried out face-to-face by the researcher in Hindi (the local language). Audio-recording of qualitative interviews were done with the permission from the respondents. Informed ethical consent from all respondents was taken before interviews with complete assurance for privacy and confidentiality.

Quantitative data were analyzed through SPSS computer software version 18. The abortion incidence was calculated in the form of percentage of women who had a history of induced abortion ever and in the past five years. Further, abortion rate was computed as the number of induced abortions occurred per 1000 pregnancies in past five years whereas abortion ratio denoted as the number of induced abortions per 1000 live births in past five years. Data related to the abortion related variables and contraception behavior of women was analyzed using descriptive statistics. Qualitative data were transcribed and translated into English and then coded and analyzed using Atlas-Ti software version 5.0.

## Results

A total of 282 women within reproductive age group were interviewed out of which 57 had an abortion history in past five years. The respondents’ mean age was 27.4 years with about 94 percent of them practicing Muslim religion. A little less than half of the women (46.8%) were illiterate with the mean years of education being 3.77 years. Similarly, 43.6 percent of respondents’ husbands also had received no education. All respondents were classified into five equally divided quintiles from poorest to richest with each category containing 20 percent of the sample. The mean per-capita monthly income was $22 while only 10.6 percent women were employed. About 71.2 percent respondents had two or more living children and 48.2 percent had more than one son. Higher proportion of older age women (38.6%) reported undergoing induced abortion than younger women (24.6%; p < 0.05). (Table 1) 

About 63 induced abortions were reported by 57 women (20.2%) in the past five years. The abortion rate was calculated to be 115.59 induced abortions per 1000 pregnancies while abortion ratio was 162.79 induced abortions per 1000 live births in past five years. About 10.5 percent of women with abortion history reported multiple induced abortions in past five years. Some of them reported to become pregnant after a very short gap of abortion which compelled them to undergo another abortion. 

The reason behind these close proximity of pregnancies and abortions were reported to be lack of awareness about health precautions in post-abortion period, careless attitude of the husbands about their wife’s health condition as well as non-use of any contraceptive methods.

About 45.6 percent women with abortion history used abortion as a means of birth spacing as they were not ready for a child at that time. Another 26.3 percent of them had undergone abortion to limit family size as they did not need any more children. Besides these affordability concerns (9.6%) and maternal health problems (7.1%) were also reported as the causes of inducing abortion.

All the 57 women with abortion history had undergone abortion within first trimester of pregnancy. Of these, 32 women (56%) had attempted medical abortion and 2 women (3.5%) traditional methods in the first instance. Rest of 23 women (40%) used surgical method of abortion. Approximately, one third (31%) of women who attempted medical abortion and half (50%) of women who opted for traditional methods initially had to seek surgical method due to incomplete abortion process. 

About 62 percent women with abortion history had sought advice for termination of pregnancy from unqualified private abortion providers in the area popularly accepted as general practitioners (GPs). Majority of surgical abortions (74%) were also performed by unregistered private facilities based medical practitioners. About 19 percent of women had brought abortion pills directly from the nearby pharmacy store without any consultation with a medical practitioner. Friends and neighbors were reported to be the primary source of advice and assistance for majority of women (53.6%) opting for medical abortion. Some women in the area were reported to be providing assistance to women in need of access to abortion medication. 

Traditional methods of inducing abortion were also reported to be attempted by few women (3.5%) such as eating ginger, cloves and raw papaya which are locally believed to result in abortion. Further, some women adopted multiple methods of abortion in one pregnancy till they succeeded. 

Several reasons were quoted by the respondents for non-use of government health services for abortion such as need for confidentiality (23%) and longer duration of stay in government hospitals (19%). Higher number of required visits, distance of facility, poor care and discomfort were other impediments for seeking government health services for abortion.

Another important reason among these women to avoid government health facility was due to rules of insisting sterilization following abortion (18%). Government health staff was reported to put pressure on the women to opt for either sterilization or insertion of copper-T following abortion services. 

The decisions regarding abortion were mostly taken by the women themselves. About 56 percent of the abortion decisions were made by the women, 7 percent by the husband and 37 percent by both partners. Approximately 19 percent women said that they did not receive consent from their husband for abortion, so they took the help of other friends and neighbours to seek abortion in a secretive manner. Even when there was approval from husband and extension of monetary support, women said there was practically no physical and emotional support from them.

Around 40 percent induced abortions were reported to have some post-abortion complications such as incomplete abortion (17%), excessive bleeding (9%), severe abdominal pain (5%), foul smelling discharge (5%) and confinement to bed and restricted daily activities (4%). The abortion related morbidities were reportedly higher (53%) with medical abortion than surgical abortion (22%). 

A majority of the 282 women (79%) were aware about at least one contraception method. However, contraception usage was low overall (41%). The use of contraception was higher among women with abortion history (47%) than among women without abortion experience (40%). Only 2 percent women opted for undergoing female sterilisation whereas no male sterilization was reported in the area. About 53 percent women were not using any contraception even after undergoing abortion. 

About 30 percent women (n = 282) stated that they feared side effects of contraception use while 14 percent of women mentioned objection of their husband. Other barriers for use of contraception were lack of awareness, pregnancy during post-partum amenorrhea and religious beliefs. 

**Table 1:** Demographic and socioeconomic characteristics of respondents

**Characteristics**	**All Respondents** **% (n = 282)**	**Women with History of Abortion ** **% (n = 57)**	**P** ** value**
Religion			
Muslim	91.1	91.2	0.608
Hindu	8.9	8.8	
Age (years)			
< 25	39.4	24.6	0.025
25-30	33.0	36.8	
> 30	27.4	38.6	
Education			
Illiterate	46.8	54.4	0.128
Literate	53.2	45.6	
Husband’s Education			
Illiterate	43.6	38.6	0.241
Literate	56.4	61.4	
Employment			
Employed	10.6	10.5	0.597
Unemployed	89.4	89.5	
Income Category			
1 (Poorest)	20	12.3	0.339
2	20	29.8	
3	20	14.0	
4	20	29.8	
5 (Least poor)	20	14.0	
Number of Living Children			
0-1	28.7	24.6	0.120
2	17.7	10.5	
> 2	53.5	64.9	
Number of Living Sons			
0	19.5	12.3	0.180
1	32.3	29.8	
> 1	48.2	57.9	
Total	100	20.2	

Reasons behind not undergoing sterilization were asked to 166 women (excluding 39 women who had already undergone Tubectomy and 79 women who had less than 2 living children). Around half (49%) of these 166 women stated that sterilization is against their religious belief. Objections of their husband and other family members and fear of operation were other factors for not undergoing sterilization.

## Discussion

High occurrence and repeated abortion practice was found to be an important health concern in this low-income slum community of Mumbai. One fifth or 20 percent women respondents had undergone induced abortion in the past five years which is approximately double of the findings of a study conducted in 2001-02 (17). Further, the rate of induced abortion was 115.6 in the study area as compared to 45.4 in Maharashtra state per 1000 pregnancies while the abortion ratio was found to be 162.79 as compared to 50.7 per 1000 live births in Maharashtra state during 1996 to 2000 (17). This difference in incidence of induced abortion might be due to the vast difference in the socioeconomic status of the study area from entire Mumbai as well as the time gap between the two studies.

Although no woman in this study reported second trimester abortion, a majority of them opted for home-based medical abortion. Many women took advice from nearby pharmacy stores or from friends and neighbors without consulting any medical practitioner. Further, the proportion of women seeking advice from the unqualified private practitioners was also very high (7,19,20-22). These pathways adopted by women are deemed unsafe and likely to cause life-threatening complications. Attempts to induce abortion using homemade concoctions were also made by some women in this community which are also practiced in other parts of India (14). Majority of surgical abortion were undertaken at the unregistered private nursing homes in and around study site which are deemed risky and unsafe (20,23-25). 

Majority of decisions for undergoing abortion were taken by women (7) which was either opposed or not supported by their husband and family members. This strong resistance from partner and family further compelled these women to secretly attempt risky methods of abortion from unskilled providers (7,26). Husband’s role was found to be narrow for providing physical assistance during hospital visits and many reported that husbands did not take precautions related to sexual behavior in the post-abortion period. This could be one of the possible reasons for frequent pregnancies and abortions with low spacing. 

Further, low levels of contraception usage reported in the study area contributed to high incidences of abortions (4). Even after undergoing one or more abortions, many women were still not using any contraception method similar to the findings of another study (16) which makes them highly vulnerable to further unintended pregnancies and abortions. This indicates poor or no post-abortion contraceptive counseling received by these women from the health providers. On the contrary, insistence and conditionality were put forth to accept long-term contraception at government health facilities discourages them to avail abortion services which in turn promotes higher number of women opting for unsafe modes of abortion from unskilled providers. High prevalence of misinformation and fear were found to be persisting among the women about the temporary contraception methods such as contraceptive pills and inter-uterine device (IUDs) (1). In addition to the strong religious beliefs against permanent family planning methods in the community, opposition from husband and family members generated strong barriers for these women to consider family planning (3,5). 

Widespread illiteracy and poor awareness among the residents of this slum community further contributed to low contraception usage, lack of preventive and precautionary measures and strong religious mis-beliefs. All these lead to a number of unintended pregnancies and abortion needs for these slum dwelling women. Further, lack of adequate qualified human resource and registered facilities for abortion services in this urban slum locality also contribute to large number of unsafe abortions. Abortion being a risky process for women’s health, repeated practice of unsafe abortions in close succession poses great danger to their health and life.

***Limitations of the Study***

The findings of this study were based on the self reported data of the respondents and therefore the estimates of abortion incidence in the area may vary from the exact values. Besides, the results are limited to only married women and may not be representative of other slum areas in India due to variation in community composition.

## Conclusion

This paper draws attention to the high number of unsafe abortions in the unorganized slum community of Mumbai city where women’s health and life are at stake. To address to this critical issue, safe abortion services can be expanded with the inclusion of practitioners of Ayurveda or Homeopathy to provide abortion care (27). Studies have also confirmed that mid-level health providers including nurse-midwives and community health volunteers can be effectively used with adequate training to provide medical abortions, first-trimester vacuum abortions and to treat incomplete abortions (27-30). Moreover, consistent awareness generation activities involving influential community leaders and couple counseling sessions with adequate options to choose culturally-acceptable contraception method can be suggested as potentially effective strategies to tackle this crucial health concern.
